# Designing small organic non-fullerene acceptor molecules with diflorobenzene or quinoline core and dithiophene donor moiety through density functional theory

**DOI:** 10.1038/s41598-021-97662-0

**Published:** 2021-10-04

**Authors:** Ghulam Bary, Lubna Ghani, Muhammad Imran Jamil, Muhammad Arslan, Waqar Ahmed, Anees Ahmad, Muhammad Sajid, Riaz Ahmad, Duohui Huang

**Affiliations:** 1 Faculty of Science, Yibin University, Yibin, 644000 Sichuan China; 2 Department of Bionanotechnology, Hanyang University, Ansan, 155-88 Korea; 3 Zhejiang Provincial Key Laboratory of Advanced Chemical Engineering Manufacture Technology, College of Chemical and Biological Engineering, Zhejiang University, Hangzhou, 310027 China; 4 Chemistry Department, Quaid-i-Azam University, Islamabad, 45320 Pakistan; 5 Faculty of Materials and Chemical Engineering, Yibin University, Yibin, 644000 Sichuan China

**Keywords:** Chemistry, Energy science and technology, Engineering, Materials science, Physics

## Abstract

The non-fullerene acceptors **A1**–**A5** with diflourobenzene or quinoline core (bridge) unit, donor cyclopenta[1,2-b:3,4-b′]dithiophene unit and 2-(2-methylene-3-oxo-2,3-dihydro-1H-inden-1-ylidene)malononitrile as acceptor unit with additional phenyl, fulvene or thieno[3,2-d]pyrimidinyl 5-oxide groups have been designed through DFT calculations. The optimization of molecular geometries were performed with density functional theory (DFT) at B3LYP 6-31G (d,p) level of theory. The frontier molecular orbital (FMO) energies, band gap energies and dipole moments (ground and excited state) have been calculated to probe the photovoltaic properties. The band gap (1.42–2.01 eV) and dipole moment values (5.5–18. Debye) showed that these designed acceptors are good candidates for organic solar cells. Time-Dependent Density Functional Theory (TD-DFT) results showed λ_max_ (wave length at maximum absorption) value (611–837 nm), oscillator strength (*f*) and excitation energies (1.50–2.02 eV) in gas phase and in CHCl_3_ solvent (1.48–1.89 eV) using integral equation formalism variant (IEFPCM) model. The λ_max_ in CHCl_3_ showed marginal red shift for all designed acceptors compared with gas phase absorption. The partial density of states (PDOS) has been plotted by using multiwfn which showed that all the designed molecules have more electronic distribution at the donor moiety and lowest at the central bridge. The reorganization energies of electron (**λ**_**e**_) (0.0007 eV to 0.017 eV), and the hole reorganization energy values (0.0003 eV to − 0.0403 eV) were smaller which suggested that higher charged motilities. The blends of acceptors **A1**–**A5** with donor polymer **D1** provided open circuit voltage (V_oc_) and ∆HOMO off-set of the HOMO of donor and acceptors. These blends showed 1.04 to 1.5 eV values of V_oc_ and 0 to 0.38 eV ∆HOMO off set values of the donor–acceptor bends which indicate improved performance of the cell. Finally, the blend of **D1**–**A4** was used for the study of distribution of HOMO and LUMO. The HOMO were found distributed on the donor polymer (**D1**) while the **A4** acceptor was found with LUMO distribution. Based on λ_max_ values, and band gap energies (E_g_), excitation energies (E_x_), reorganization energies; the **A3** and **A4** will prove good acceptor molecules for the development of organic solar cells.

## Introduction

Presently, due to high demand of the energy and depletion of non-renewable resources, there has been great deal of focus on the alternate energy sources. The great advancement in the development of solar cells have been made^1^. Among various types of solar cells, the organic solar cells (OSCs) have received great attention due to their easy process-ability, low cost, tuning of the Highest Occupied Molecular Orbitals (HOMO) and Lowest Unoccupied Molecular Orbitals (LUMO). In organic solar cells, usually a polymer is used as donor material along with well-known fullerene as an acceptor to build heterojunction between donor and acceptor. The fullerene and its derivatives have been an immense choice for the chemists as small molecular acceptor (SMA) due to high power conversion efficiency (PCE), electron transport in three dimensions, high charge separation and low lying LUMO^2^. Therefore, lots of efforts have been devoted to develop OSCs with polymer-fullerene bulk heterojunctions (BHJ)^3–5^. The PCE of these OSCs with fullerene derivatives have exceeded 12%^6–10^. However, the disadvantages of the fullerenes such as fixed energy levels, solubility in organic solvents, poor processability, and poor light absorption above 600 nm created space for the development of alternative acceptor molecules^11–15^. The small organic molecular non-fullerene acceptors (FNAs) have been found an alternative to fullerene that removed the disadvantages associated with fullerene acceptor. These molecules were found to be easily fabricated through solution processed BHJ, and improved the film properties of the organic solar cells. The NFAs were designed with structural variation which considerably improved the PCE and film morphology of the OSCs. This structural variation also proved helpful in constructing the molecules with desirable HOMO and LUMO energies and optical band-gap energy. No doubt these small NFAs proved to be superior to the fullerene counterpart as these have removed the issues associated with fullerene derivatives, such as these have tunable frontier molecular orbitals (FMOs), good solubility in organic solvents, wide absorption band in visible region and transparency in the fabricated film^14–16^. A great deal of effort has been devoted in designing the NFAs for photovoltaic applications. The 3a,3b,6a,7a-tetrahydro-7H-cyclopenta[1,2-b:3,4-b′]dithiophene as donor moiety has been frequently used within the structure of NFAs due to its easy availability and good compatibility with a number of acceptor moieties. These properties of the donor moiety was found to be due to the efficient electron-donating nature of sulfur atom in acceptor–donor–acceptor (A–D–A) type structure of NFAs^17–19^. The fluorine substituted organic small molecules as photovoltaic materials offered promising results when these were fabricated in solar cells^20^.

The density function theory (DFT) has recently been used for the designing of photovoltaic materials. Isoindigo-dithiophenepyrrole based D–A oligomers were successfully designed by Ahmed et al*.* through DFT and time dependent density function theory (TD-DFT)^21^. Mehboob et al*.* designed acceptor molecules with benzodithenophene core and melanonitrile or dinitromethane electron–acceptor end groups. These designed molecules showed high charge mobility and low band-gap values at B3LYP level of theory with split valence 6-31 G(d,p) basis-set^22^. Farah et al*.* calculated the optical and electronic properties of newly designed dithieno[3,2-b:2′,3′-d]silole)2,6-diyl (DTS) based donor molecules through DFT and TD-DFT at CAM-B3LYP/6-31G (d) level of theory. FMOs energy, V_oc_, reorganization energies, excitation energy of the designed molecules were found better than the reference molecules^23^. Farhat et al*.* in 2020 used DFT at B3LYP with 6-31G(d,p) basis-set to calculate the λ_max_, reorganization energy (charge mobility), frontier molecular orbitals (FMOs) energy and open circuit voltage (V_oc_) of newly designed subphthalocyanine derived chromophores as donor materials. These designed donor molecules showed improved photovoltaic properties than the reference compounds^24^. Thus DFT with B3LYP theory level showed promising results for the evaluation of photovoltaic materials.

Li et al*.* in 2019 successfully synthesized NFAs with diflourophenylene as core structure, cyclopenta[2,1-b:3,4-b′]dithiophene donor and malanonitrile end-capped acceptor groups. The fabrication of these acceptors with the donor polymers provided good photovoltaic properties such as small E_loss_ and high PCE of above 10%^25^. By inspiring from the work of Li et al*.*, acceptor molecules have been designed with diflourophenylene or quinoline core, cyclopenta[1,2-b:3,4-b′]dithiophene as donor unit and melanonitrile based acceptor units that provided enhanced photovoltaic properties through DFT calculation. These types of molecules were developed, and studied for photovoltaic properties. However, the power conversion efficiency of these molecules reached only 10%. The current work, herein, presents the designing of the NFA molecules with enhanced photovoltaic properties, and it is expected that these designed FNAs will improve the PCE of organic solar cell greater than 10% when will be fabricated in OSCs.

## Computational detail

All the geometry optimizations were carried out by using Gaussian 09 package^26^ with Gaussview 5.0^27^ for viewing the molecules. For geometry optimization, first of all the acceptor molecule **A1** which has closed similarity with standard^25^ was optimized through DFT with a split valence basis set 6-31G (d,p) that is used at B3LYP^28^ and CAM-B3LYP^28^ levels of theory. It was noted that the B3LYP/6-31G (d,p) gave results of energy gap (E_g_) close to the experimentally determined values for structurally similar NFA compound^25^. Therefore, B3LYP/6 31G (d,p) was used for the geometry optimization of designed acceptor molecules **A1** to **A5**. The energies of the highest occupied molecular orbital (HOMO), lowest unoccupied molecular orbitals (LUMO) and band gap energies (E_HOMO_–E_LUMO_) were calculated at the same level of theory. The optoelectronic properties such as λ_max_, oscillation factor (*f*) and excitation energies were calculated with time dependent self-consistent field method (TD-SCF)^29^ at the same level of theory in gas phase and in CHCl_3_ using the integral equation formalism variant (IEFPCM)^30^. The properties of the designed molecules (**A1–A5**) were studied in the dissolved form in CHCl_3_. The solution processed fabrication of the solar cells for film formation is usually done with CHCl_3_ as the solvent of choice due to its good dissolution good properties. The density of states, dipole moments and reorganizational energies (**λ**) for electron and hole were calculated at the B3LYP 6-31 G (d,p). The Gaussam software^31^ was used for the simulation of the absorption spectra that were plotted by using MS excel. The reorganization energy can be associated with external sphere such as selection of medium or electron-transfer processes. However, the internal reorganizational energy is associated with the reorganization of geometric parameters. The internal reorganizational energies of electron (**λ**_e_) and hole (**λ**_h_) have only been focused and, thus calculated by using the following equations.

1 $${{\varvec{\uplambda}}}_{\mathrm{h}} = ({\mathrm{E}}_{\mathrm{o}}^{+}- {\mathrm{E}}_{+}) + ({\mathrm{E}}_{+}^{\mathrm{o}}- {\mathrm{E}}_{\mathrm{o}})$$

2 $${{\varvec{\uplambda}}}_{{\varvec{e}}} = ({\mathrm{E}}_{\mathrm{o}}^{-}- {\mathrm{E}}_{-}) + ({\mathrm{E}}_{-}^{\mathrm{o}}- {\mathrm{E}}_{\mathrm{o}})$$

The E^o^_+_ and E^o^_−_ correspond to the energies of cation and anion with neutral geometry; whereas, E_+_ and E^−^ are the energies of cation and anion. The E_o_ is energy of the molecules at their neutral state. Density of the states (DOS) and partial densities of states (PDOS) have been extracted from the formatted check Gaussian files using multiwfn^32^.

## Result and discussion

**Figure 1:**
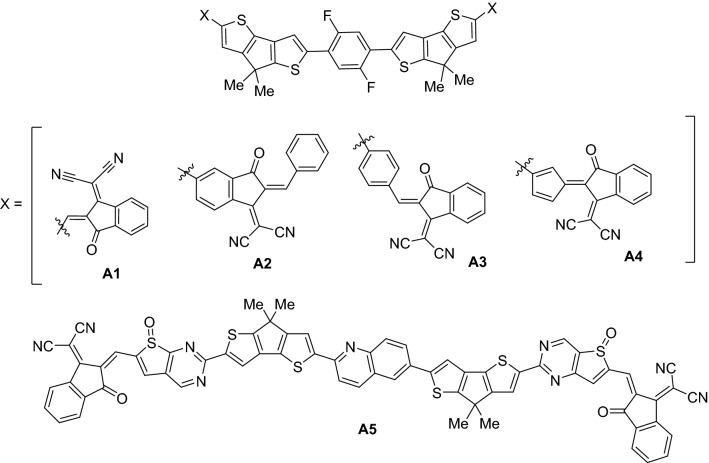
The structure of designed small organic NFA molecules **A1**–**A5**; the topmost structure represents core and donor units with X as acceptor unit (**A1–A4**), the lowest **A5** has core, donor and acceptor units.

By taking inspiration from the work of Li et al*.,* the small organic molecular NFAs **A1**–**A5** have been designed with cyclopenta[1,2-b:3,4-b′]dithiophene donor moiety (Fig. 1) through DFT calculations. The **A1** has close similarity with the small organic NFA developed by Li et al*.* except the cyclopenta[2,1-b:3,4-b′]dithiophene^25^ donor moiety was replaced with cyclopenta[1,2-b:3,4-b′]dithiophene. The acceptor moieties of the designed molecules **A1**–**A3** have been derived from 2-(2-methylene-3-oxo-2,3-dihydro-1H-inden-1-ylidene)malononitrile, but **A4** has fulvene group in addition to 2-(2-methylene-3-oxo-2,3-dihydro-1H-inden-1-ylidene)malononitrile end capped acceptor group. The **A1**–**A4** molecules have diflourophenylene as bridge structure. The designed **A5** molecule has qunioline core structure and the acceptor moiety contains thieno[3,2-d]pyrimidinyl 5-oxide incorporated in between donor cyclopenta[1,2-b:3,4-b′]dithiophene and end-capped cyclopenta[1,2-b:3,4-b′]dithiophene group (Fig. 1). The goal of the present work is to design NFA molecules with improved photovoltaic properties. The already developed NFAs showed only 10% PCE in organic solar cells, but the present work has focused the designing new NFAs which will show significantly improved photovoltaic properties such as low excitation energy, low optical band-gap, more absorption, high dipole moment and low reorganizational energies. These theoretically calculated parameters would lead to develop molecules with improved properties in a solar cell.

### Optimized geometries and frontier molecular orbitals (FMOs)

The study of the FMOs is useful for the optical and electronic properties of designed molecules **A1**–**A5**. These designed molecules were optimized by using density functional theory at B3LYP 6-31G (d,p).

Table 1 shows the HOMO, LUMO energies and band gap energies of **A1** to **A5** molecules. The **A2** molecule has the lowest energy of the HOMO (− 5.15 eV) while the **A4** has highest energy of HOMO (− 4.39 eV). The band gap energies (E_g_) are considered as an important parameter as lower E_g_ values make excitation easier, thus improves the photoexcitation process. The designed molecules **A3** and **A4** have lowest E_g_ values (1.42 eV), **A2** has highest E_g_ value (2.01 eV) among the designed acceptors, and the E_g_ of **A1** and **A5** were calculated to be 1.80 eV and 1.66 eV respectively. The ΔSCF based energy of HOMO–LUMO band-gap (E_g_) of **A4** was also calculated to make sure that the approximation of quasi-particle orbitals as their Kohn Sham values are valid for these systems. The energy of neutral, cationic and an anionic state of the **A4** molecule were calculated which were followed by the calculation of the vertical ionization potential (IP) and electron affinity (EA). Finally, the difference of IP and EA provided ΔSCF based band gap energy (1.47 eV). The values of HOMO, LUMO and Eg with DFT/B3LYP and ΔSCF are close to each other for **A4** molecule which approximates that the given basis set is valid and has provided good results for these molecules (Table 1).

**Table1:** The calculated orbital and band gap energies of **A1**–**A5** at B3LYP 6-31 G(d,p) in gas phase.

Molecules	E_HOMO_ (eV)	E_LUMO_ (eV)	E_g_ (eV)
**A1**	− 4.77	− 2.97	1.80
**A2**	− 5.15	− 3.14	2.01
**A3**	− 4.39	− 2.97	1.42
^a^**A4**	− 4.42	− 3.00	1.42 (1.47)
**A5**	− 5.09	− 3.43	1.66

^a^The value E_g_ for **A4** in parenthesis have been calculated by using ΔSCF/B3LYP.

The distribution of HOMO and LUMO in acceptor molecules gives information on the delocalization of electronic density. The HOMO describes the valence band, while the LUMO gives information about the conduction band of acceptor molecules. The band-gap energy is directly related to the structure of the molecule. The lowing of band-gap energy is related to the extended conjugation with more planar geometry of the molecule. The **A3** and **A4** molecules have planar geometry and extended conjugation due to phenyl or fulvene rings between donor and acceptor moieties, thus these molecules showed lowest E_g_ value (1.42 eV). However, the direct attachment of the acceptor moiety with the donor moiety in **A1** and **A2** showed increase E_g_ values (1.80 and 2.01 eV respectively) which can be associated with the reduced conjugation in the molecules. Finally, **A5** which has quinoline bridge showed intermediate value of the Eg (1.66 eV). The **A1**–**A4** molecules have cyclopenta[1,2-b:3,4-b′]dithiophene donor diflourophenylene as bridge structure. The designed **A5** molecule has qunioline core structure, and the acceptor moiety contains thieno[3,2-d]pyrimidinyl 5-oxide incorporated in between donor cyclopenta[1,2-b:3,4-b′]dithiophene and end-capped cyclopenta[1,2-b:3,4-b′]dithiophene group.

**Figure 2:**
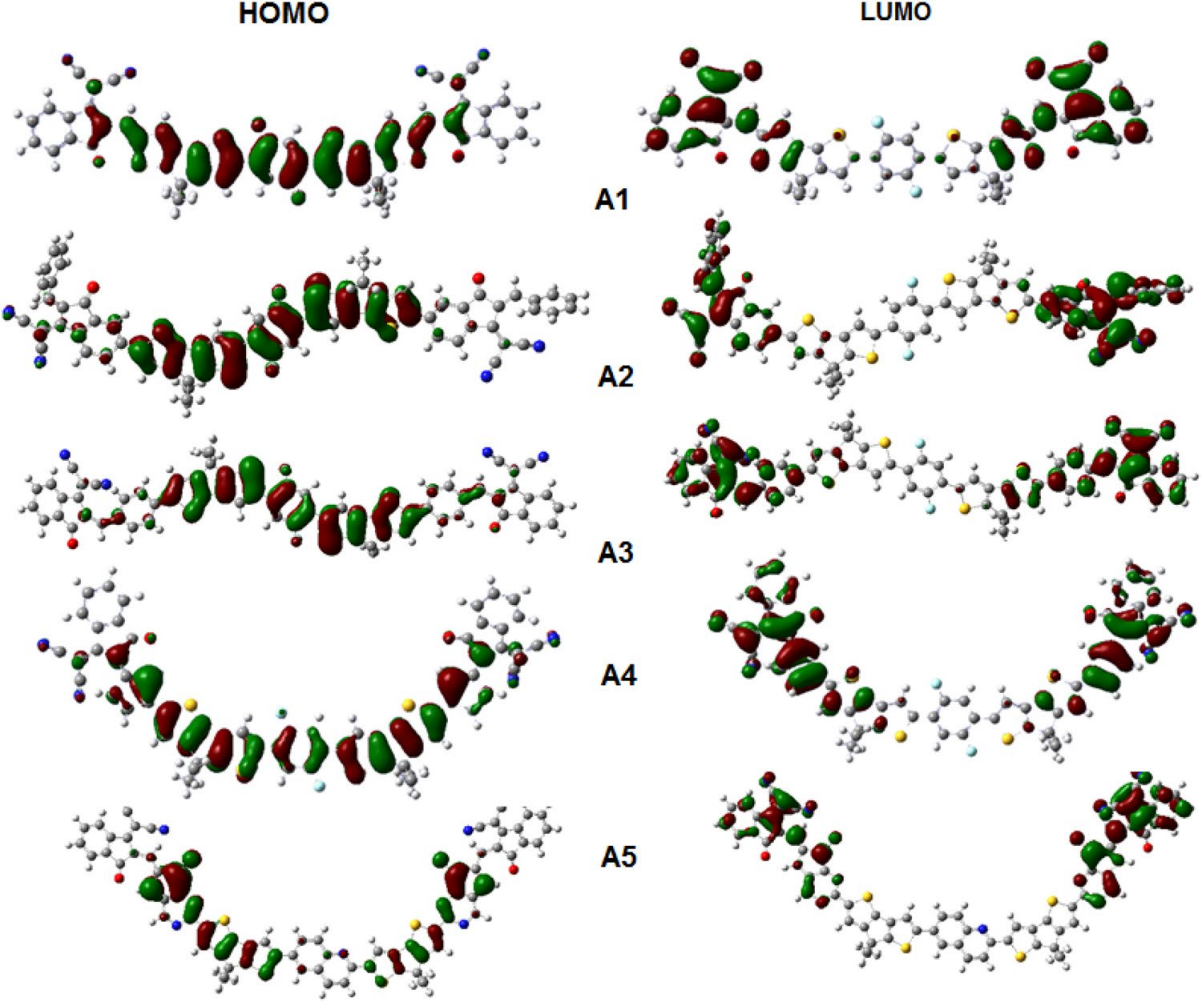
Distribution of HOMO and LUMO of the molecules **A1**–**A5** at B3LYP 6-31 G (d,p).

Figure 2 shows that the **A1**–**A4** has their HOMO distributed over the bridge and donor moieties. It can be visualized that the **A1–A4** has HOMO distribution on the bridge and donor units, while **A5** has HOMO distribution on the donor moiety. The LUMO distribution for all the designed molecules has resided on the acceptor units.

The further confirmation of FMOs has been achieved through study of the partial density of the states (PDOS) analysis for all the designed **A1-A5** molecules. The designed molecules were divided in to three fragments; acceptor which corresponds to the end-capped groups, donor fragment which consists of 7,7-dimethyl-7H-cyclopenta[1,2-b:3,4-b′]dithiophene and bridge structure which consist of difloropheylene for **A1**–**A4** or quinoline unit for **A5**.

`

**Figure 3:**
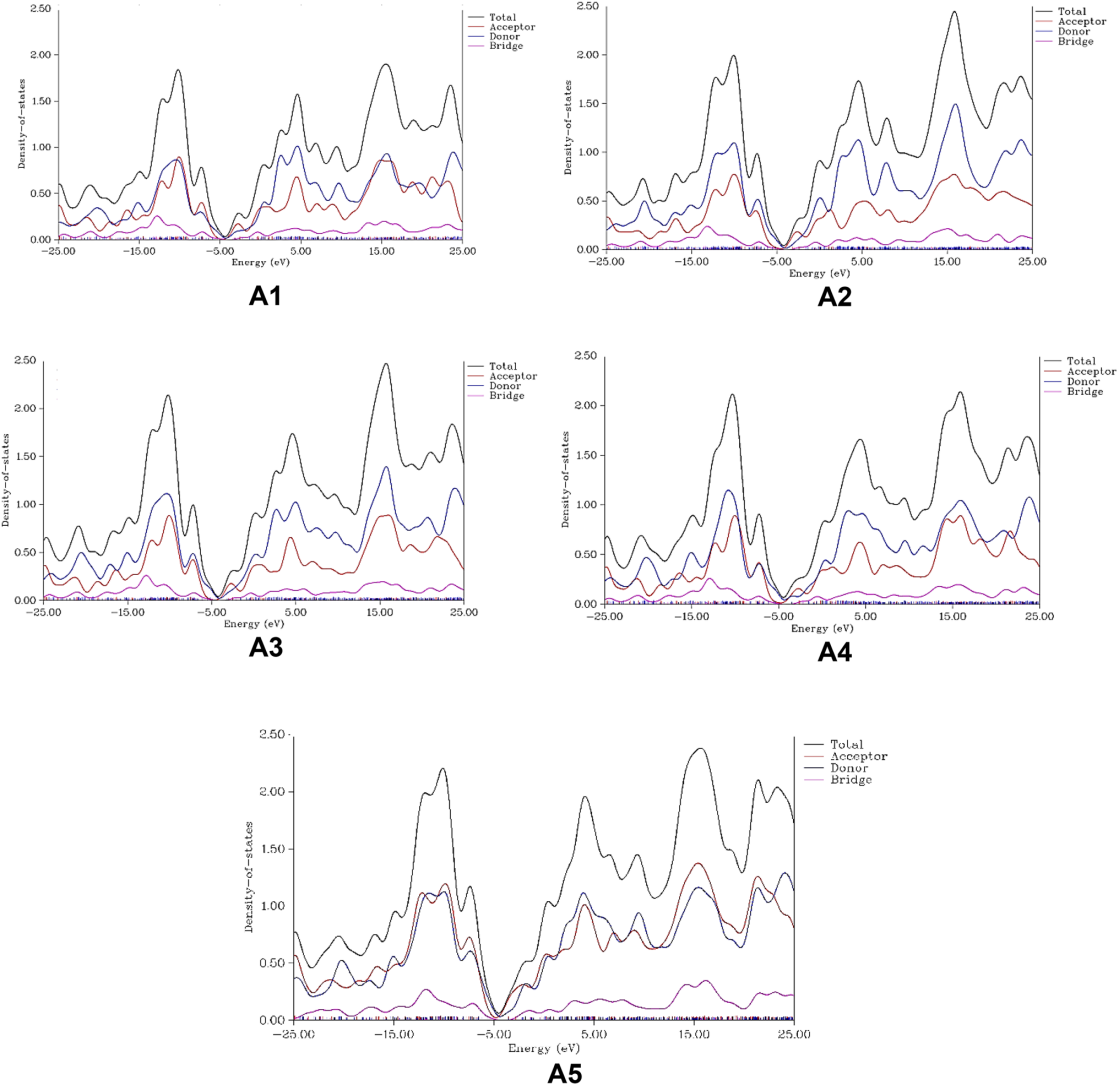
Total and partial density of states (PDOS) calculated at B3LYP/6-31G(d,p) for the designed molecules **A1**–**A5**.

The distribution of total density of states is represented in Figure 3 by black line, donor units with blue lines, acceptor units with red lines and the bridge groups with green lines. The density distribution along negative x-axis values represent valence band (HOMO) and the distribution along positive x-axis value shows conduction band^32^. Figure 3 shows that the distribution of electron densities lies more on donor moieties and less on acceptor moieties; however, near the fermi energy, the acceptor unit (red curve) in all **A1–A5** molecules have considerable contribution of density of states (DOS) in molecular orbitals. This trend suggests that these type of molecules can contribute effectively as acceptor molecules when fabricated in solar cells; and can, presumably, enhance the efficiency of the organic solar cell. The knowledge of the DOS is crucial for enhancing the organic solar cell efficiency. The DOS knowledge can lead to the better understanding of the carrier mobilities of the NFA molecules in a fabricated film^33^. The DOS of the blended film with HOMO (valence band) of donor and LUMO (conduction band) of the acceptor (NFAs in present case) molecules are considered. The DOS around the LUMO of NFAs are important, especially the PDOS on the acceptor moiety of FNAs. The more PDOS on the acceptor moiety suggests good electron affinity of the acceptor which can provide enhanced photovoltaic properties to the OSCs. All the designed molecules exhibit the more space of the PDOS on the conduction band (LUMO) than the valence band (HOMO) which suggest that these can accommodate excited electron efficiently^34^. The Bridge of all the designed molecules has lowest distribution. For **A1,** the donor and acceptor moieties have about equal distribution in the valence band region, whereas in conduction band region, the acceptor surpasses the donor moiety when moving to the positive on x-axis. Similar trend is also exhibited by the **A5** molecule. The **A2**–**A4** molecules exhibit more contribution of donor than acceptor units (Fig. 3).

### Dipole moments of designed acceptor A1–A5 molecules

The dipole moments of the designed **A1**–**A5** molecules with optimized geometry (µ_g_) and the excited state (µ_e_) were calculated at B3LYP 6-31G (d,p) level of theory in gas phase. Table 2 shows the µ_g_, µ_e_ and µ_e−_ µ_g_ values. The dipole moment is an important parameter to evaluate the NFA molecules. The compounds with higher dipole moment can have higher charge separation. This higher charge separation results in the improved capability of the molecule to accommodate  the excited electrons. The high dipole moment also favours the easy fabrication of the material film through solution based bulk-heterojunction due to their high solubility in organic solvents^35^. The higher values of µ_e_ have been calculated for **A5** and **A1** with 18.9 D and 13.8 D respectively. Similarly, the µ_g_ for **A5** is 12.8 D and for **A1** is 6.4 D respectively**.** The µ_e_–µ_g_ for **A1** is 7.4 D and 6.1 D for **A5**. The higher values of µ_e_–µ_g_ show that the transition state has considerable polarity compared with ground state. The µ_e_ values for **A2**–**A4** are 9.1, 7.7 and 8.7 D respectively, and their ground state (µ_g_) values are in the range of 5.5–8.9 D. The µ_e_–µ_g_ values for **A2**–**A4** are 0.2, 1.8 and 3.2 D. The lowest value of µ_e_–µ_g_ for **A2** describes that the excited state has marginally higher polarity than the ground state geometry. Based on the dipole moment calculation, the **A5** and **A1** molecules are presumed to be more efficient for the fabrication through solution processed bulk heterojunction.

**Table 2:** The ground state (**µ**_**g**_) and excited state dipole moments (**µ**_**e**_) of **A1**–**A5** in gas phase.

Compounds	µ_e_	µ_g_	µ_e_–µ_g_
A1	13.8	6.4	7.4
A2	9.1	8.9	0.2
A3	7.7	5.9	1.8
A4	8.7	5.5	3.2
A5	18.9	12.8	6.1

### Photo-excitation of A1–A5 acceptor molecules

The photo-excitation (UV) studies were calculated at B3LYP 6-31G (d,p) with time-dependent density functional theory (TD-DFT). The optical properties were calculated in gas phase and in CHCl_3_ solvent (IEFPCM) for the designed **A1**–**A5** molecules. Table 3 shows λ_max_, excitation energies (E_x_) which correspond to the first excited state, oscillation factor (*f*) and percent contribution of HOMO to LUMO in gas phase. The value of **λ**_**max**_ shows red shift in the order **A4** > **A5** > **A1** > **A3** > **A2**. The increase in the λ_max_ can be attributed to the extended conjugation in a molecule. The extended conjugation, especially due to the bridge moiety of the small organic FNA molecules can enhance the efficiency of solar cells. The extended conjugation has been found to decrease the LUMO energy which results in the low band-gap and higher shift in λ_max_ values^36^. The red shift in λ_max_ value along with strong light absorption has resulted to lower the excitation energy, and ultimately reinforce the electron-acceptor property of the molecules^37^. The presence of fulvene extended conjugation in the **A4** acceptor molecule and resulted in highest λ_max_ value (810 nm). The quinoline core structure in **A5** is responsible for the extended the conjugation which ultimately shifted λ_max_ towards higher side (778 nm). **A1**, **A2** and **A3** have 670 nm, 631 nm and 645 nm λ_max_ values. The E_x_ value provides useful information about the excitation of an electron from valence band (HOMO) to conduction band (LUMO). Lower value of E_x_ makes excitation easier. Table 3 shows that the order of E_x_ values is **A2** > **A1** > **A5** > **A3** = **A4**. The **A2** has highest value of 1.92 eV, while **A3** and **A4** has the lowest value (1.39 eV). The acceptor molecules **A1** and **A5** have 1.72 and 1.62 eV of excitation energy values. Assignment of HOMO → LUMO is found in the order **A1** (93%) = **A3** (93%) > **A4** (90%) > **A5** (84%) > **A2** (78%).

**Table 3:** The calculated UV results of **A1–A5** at TD-DFT/B3LYP 6-31G(d,p) in gas phase.

Molecules^a^	λ_max_ (nm)	E_x_ (eV)	*f*	Assignment
A1	670	1.72	0.68	H → L (93%)
A2	631	1.92	1.34	H → L (78%)
A3	645	1.39	0.87	H → L (93%)
A4	810	1.39	0.80	H → L (90%)
A5	778	1.62	0.82	H → L (84%)

Table 4 shows the optoelectronic properties of the designed **A1**–**A5** molecules in CHCl_3_ solvent with IEFPCM model. The λ_max_ (nm) values are found in the order **A4** > **A5** > **A3** > **A1** > **A2.** The **A4** shows highest value (847 nm), and **A2** has lowest values (678 nm). The absorption values for **A1**, **A3** and **A5** are found to be 698, 706 and 789 nm. The excitation energy values for designed NFA molecules (**A1–A5**) which correspond to the first excited state have also been calculated in solvent CHCl_3_. The E_x_ values of **A3** and **A4** are 1.40 eV which are the lowest among all the designed acceptor molecules due to the extended conjugation with fulvene moiety is incorporated between donor and acceptor groups. The **A2** has the highest E_x_ value (1.88 eV), while the **A1** and **A5** have 1.70 and 1.59 eV respectively. The oscillation factor (*f*) has value range from 0.77 to 1.09 for **A1**–**A5.** HOMO → LUMO contributions are highest for **A1** with 97%, while **A3** has 95% contribution. The **A2**, **A4** and **A5** have 85–90% contributions of HOMO → LUMO (Table 4). The **A3** molecule has phenyl ring immersed between donor and acceptor moieties which increased the conjugation due to its planar structure and hence showed highest absorption value in gas and in CHCl_3_ solvent (with IEFPCM model) as compared to the other designed acceptor molecules. The extended conjugation and planarity are considered as important for increasing absorption of the radiations by the molecules^37,38^. All the designed acceptor molecules **A1–A5** showed lower value of the excitation energies (E_x_) than the ground state band-gap energies(E_g_). This trend is consistent with previously reported data of the E_x_ and E_g_ values^39^.

**Table 4:** The calculated UV results of **A1–A5** at TD-DFT/B3LYP 6-31G(d,p) in CHCl_3_.

Molecules^a^	λ_max_ (nm)	E_x_ (eV)	*f*	Assignment
A1	698	1.70	0.77	H → L (97%)
A2	678	1.88	1.46	H → L (85%)
A3	706	1.40	1.09	H → L (95%)
A4	847	1.40	1.06	H → L (90%)
A5	789	1.59	1.05	H → L (87%)

^a^Values are calculated in CHCl_3_ with IEFPCM model.

The **A4** molecule has the highest λ_max_ value in gas as well as in solution (CHCl_3_ solvent) phase. The **A4** molecule contains fulvene moiety due to which this shifted the λ_max_ to the higher side. This fulvene moiety is linear and an effective over-lap of the π-orbitals to its neighboring moieties rendered it to extend conjugation which ultimately resulted in absorption of UV in the higher wave-length range. The phenyl moiety in **A2** and **A3** could not enhance conjugation effectively as is evident from the λ_max_ values of these molecules (**A2–A3**) compared with **A1** in both gas phase and in CHCl_3_. This can be attributed to the aromaticity of the phenyl ring which may show reluctance to contribute in extending conjugation. Thus λ_max_ values could not get considerable increase toward red-shift. However, the theinopyimidine oxide moiety showed considerable trend in extending conjugation in the designed molecule **A5** and shifted the value of λ_max_ towards longer wave-length (789 nm).

**Figure 4:**
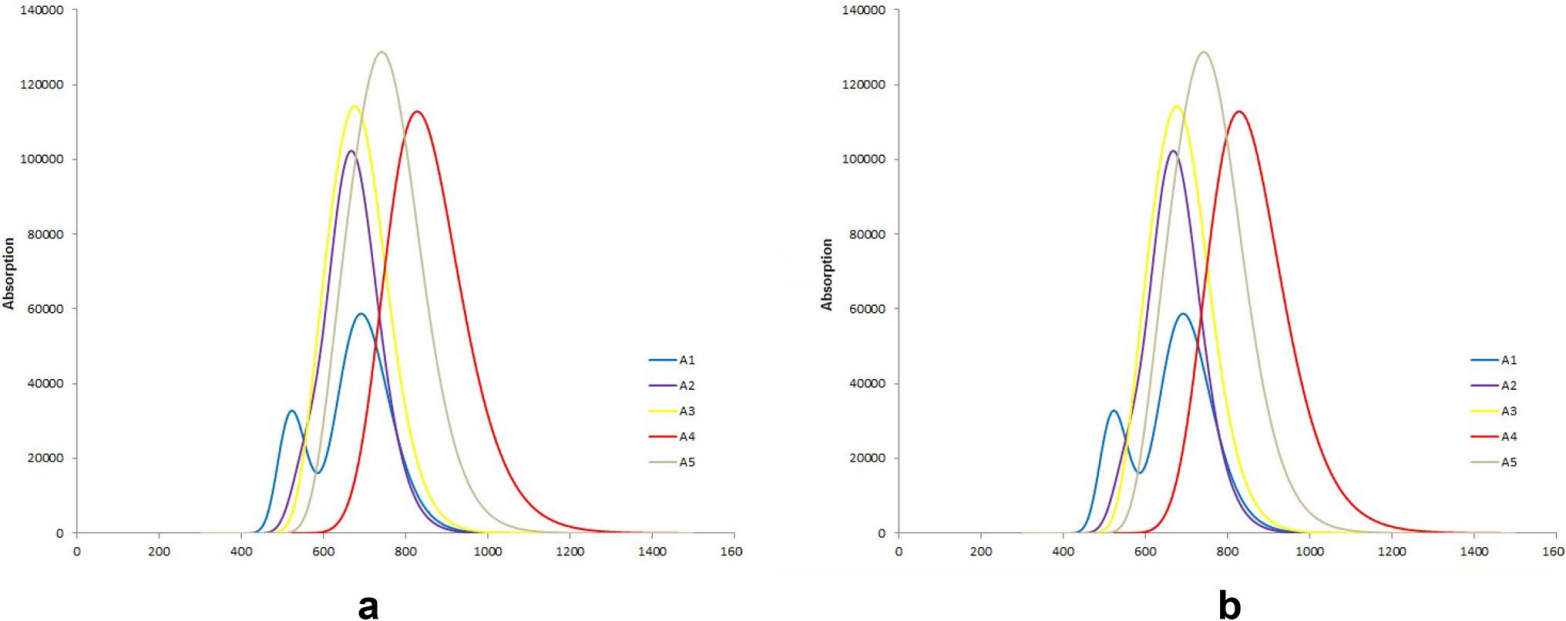
The UV absorptions of **A1**–**A5** at TD-DFT B3LYP 6-31 G(d,p): (**a**) in gas phase, (**b**) in CHCl_3_ (IEFPCM model).

Figure 4 shows the absorption spectra of the **A1**–**A5** molecules in gas phase and in CHCl_3_ solvent (IEFPCM model) with TD-DFT/B3LYP 6-31G (d,p). The similar trend can be found in gas phase (Fig. 4a) and in CHCl_3_ solvent (Fig. 4b) except λ_max_ of all the molecules shows marginal red shift in CHCl_3_ solvent. The lowest absorption was observed for designed acceptor **A1** in gas as well as in CHCl_3_. The highest absorption is given by **A3** which can be attributed to the extended conjugation due to the presence of phenyl ring. The order of the absorption values for other acceptor molecules is **A5** > **A2** > **A4** in gas phase and in CHCl_3_ (Fig. 4).

### Reorganizational energies of A1–A5 molecules

The reorganizational energies of the hole (**λ**_h_) and electron (**λ**_e_) for designed **A1**–**A5** NFA molecules are given in Table 5. The reorganizational energy is generally divided into external reorganizational energy (**λ**_ext._) and internal reorganizational energy (**λ**_int._). The **λ**_ext_ results due to the external factors, such as environment, film morphology and temperature. The **λ**_ext._ is due to the intrinsic molecular structure and is only focused here. The reorganizational energy is inversely related to the mobility of electron and hole. When the reorganizational energy is lower, the mobility of ions will be higher^40^.

**Table 5:** Reorganizational energies of **A1**–**A5** at B3LYP 6-31G (d,p).

Compounds	λ_e_ (eV)	λ_h_ (eV)
A1	0.0027	0.0099
A2	0.0077	0.001
A3	0.0007	0.0003
A4	0.0059	0.0361
A5	0.0117	0.0403

The **A3** has the lowest reorganizational energies of electron (0.0007 eV) and hole (0.0003 eV), therefore, it offers the highest mobilities of electron and hole. The **λ**_**e**_ for the rest of molecules are in the order of **A5** > **A4** > **A2** > **A1**and their mobility is in the **A1** > **A2** > **A4** > **A5** order. The order of the **λ**_**h**_ is **A5** > **A4** > **A1** > **A2** and their hole mobility order is **A2** > **A1** > **A4** > **A5**. The electron and hole reorganization energies for a molecule are also different. The **A1** and **A5** has less electron reorganizations (**λ**_**e**_) than the hole reorganization (**λ**_**h**_). But **A2** and **A3** exhibited higher **λ**_**e**_ than corresponding **λ**_**h**_ (Table 5).

### Open circuit voltage (V_oc_) and difference of HOMO of donor and acceptor

The open circuit voltage (V_oc_) is an important parameter in the fabrication of organic solar cells. When the solar cell is in zero current level, the maximum voltage drawn out of the cell is called V_oc_. The higher value of V_oc_ indicates higher fill factor (FF) which is a key parameter in determining the efficiency of solar cells. The theoretical V_oc_ value is calculated through the difference of HOMO of donor and LUMO of acceptor minus 0.3. The donor polymer **D1** was used at B3LYP 6-31G (d,p) along with **A1**–**A5** acceptors for the calculation of V_oc_ through the following equation.

3 $${\mathrm{V}}_{\mathrm{oc}}= {\mathrm{HOMO}}_{\mathrm{donor}}- {\mathrm{LUMO}}_{\mathrm{acceptor}}- 0.3$$

The theoretical calculation of V_oc_ by subtracting the HOMO of donor from LUMO of acceptor and 0.3 can provide an estimation of the open circuit voltage when the designed acceptor is fabricated with some donor polymer (**D1**) through solution processed bulk heterojunction^41,42^. The highest V_oc_ value of 1.5 eV is shown by the **A1** and **A3** with donor polymer **D1**. The blends of **A2** and **A4** with **D1** provide 1.33 eV value of V_oc_. The lowest value of 1.04 eV was calculated for **D1**–**A5** blend.

The difference of energies of HOMO of donor **D1** and acceptors **A1**–**A5** provide HOMO off-set values (∆HOMO_D–A_). The ∆HOMO_D–A_ provides important information about the efficiency of the solar cell. It has been reported that lower ∆HOMO_D–A_ value results in the enhanced solar cell efficiency^25^.

**Figure 5:**
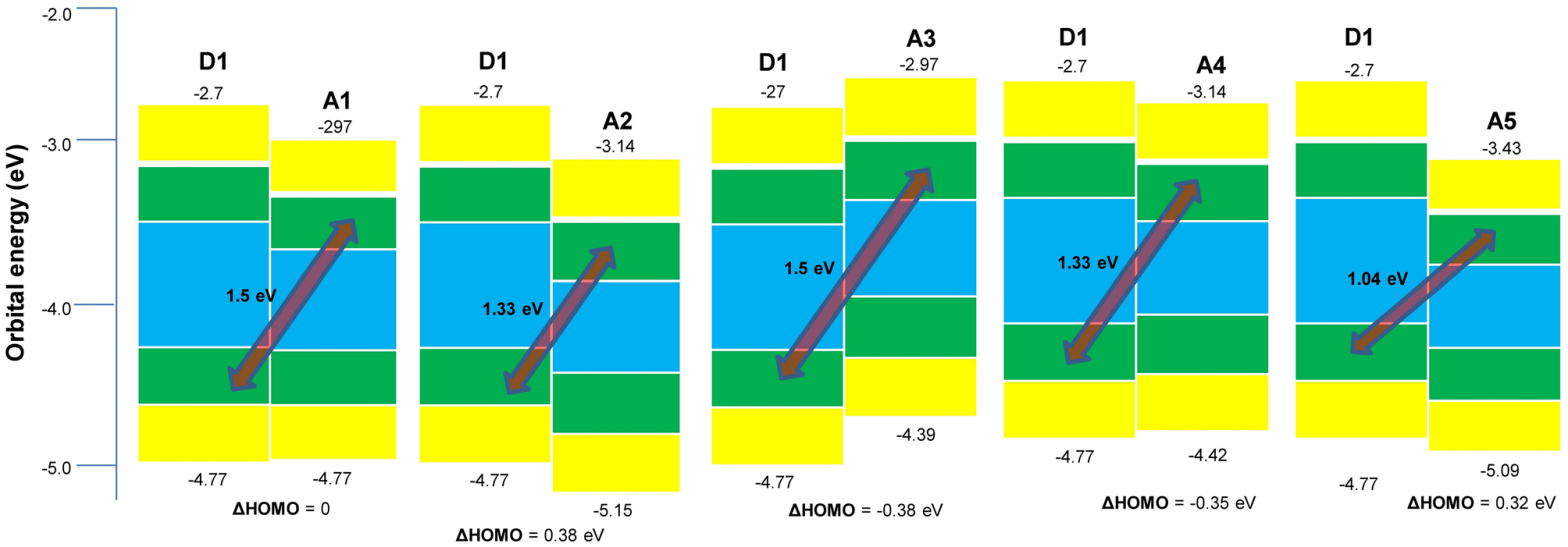
V_oc_ and ∆HOMO of **A1**–**A5** with **D1** polymer donor.

The **D1**–**A1** blend shows zero ∆HOMO_D–A_ off-set values, while the **D1–A2** and **D1**–**A4** blends provide − 0.38 eV and 0.35 eV respectively. 0.32 eV value is observed for **D1**–**A5** (Fig. 5).

**Figure 6:**
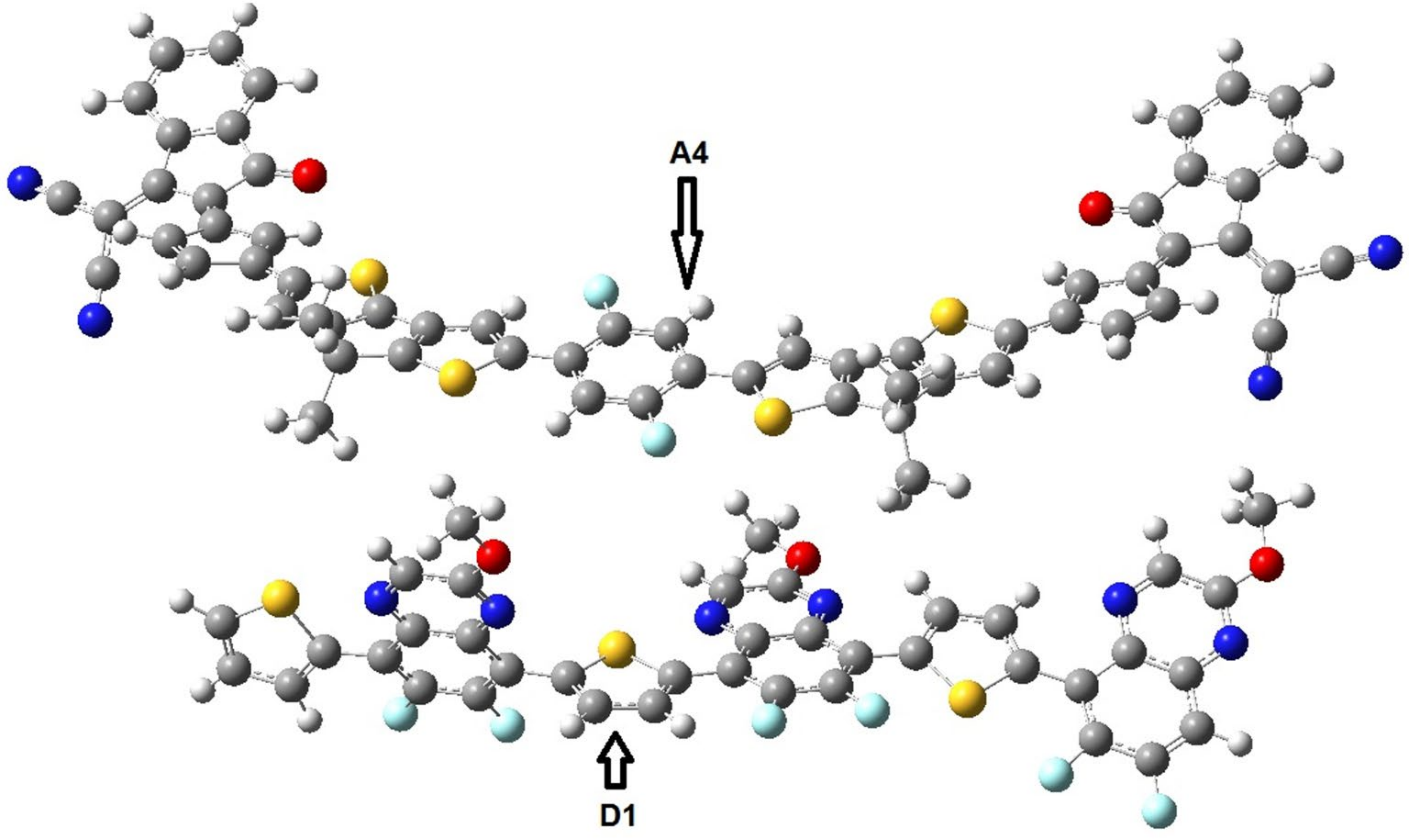
Optimized geometry of blend of **D1**–**A4** at B3LYP 6-31G (d,p).

**Figure 7:**
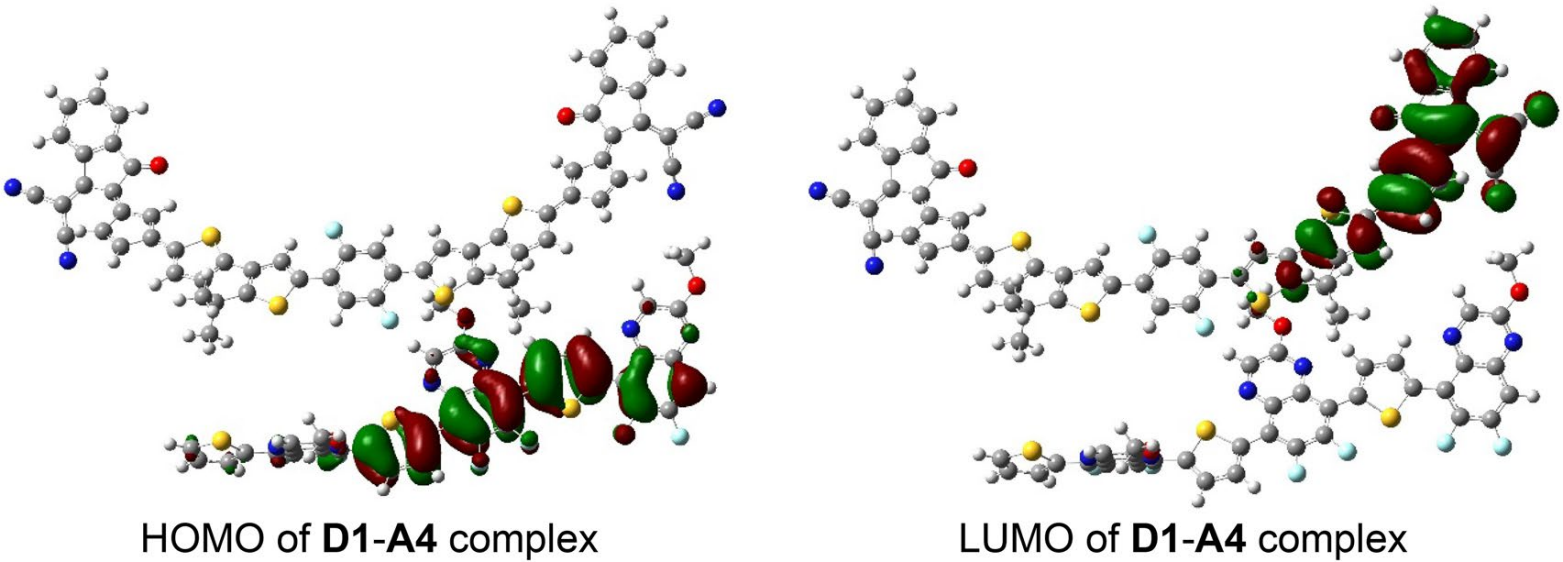
HOMO and LUMO distribution on the blend of **D1**–**A1.**

The complex of **A4** with **D1** has been used for studying charge transfer between donor and acceptor molecules (Fig. 6). Figure 7 shows that the HOMO are scattered on the donor polymer **D1** while the LUMO are entirely distributed on the acceptor **A4**. The calculation of dipole moment provides useful information about the charge transfer as well as the electrostatic interaction between donor and acceptor units^43,44^.

## Conclusion

The non-fullerene **A1**–**A5** acceptor molecules have been designed successfully with diflourobenzene or quinoline bridge, cyclopenta[1,2-b:3,4-b′]dithiophene donor and 2-(2-methylene-3-oxo-2,3-dihydro-1H-inden-1-ylidene)malononitrile acceptor moiety which incorporates additional phenyl, fulvene or thieno[3,2-d]pyrimidine 5-oxide units through DFT calculations. For the illustration of the optoelectronic, photovoltaic and structure related properties; the E_HOMO_, E_LUMO_, E_g_, λ_max_, E_x_, and PDOS has been calculated. The designed non-fullerene acceptor **A4** has shown lowest band gap energy (1.42 eV), as well as excitation energy in gas phase (1.39 eV) and in CHCl_3_ (1.40 eV). Thus **A4** will, presumably, prove good candidate for future organic non-fullerene acceptors. The dipole moments of the designed acceptors **A1**–**A5** at ground state (µ_**g**_) and excited state (µ_**e**_) have also been calculated; **A5** showed highest dipole moment values (µ_**e**_:18.6D; µ_**e**_:12.8D). The reorganizational energies of electron (λ_**e**_) and hole (λ_**h**_) transport for **A1**–**A5** are estimated. The **A4** has the lowest λ_**e**_ (0.0007 eV) and λ_**h**_ (0.0003 eV) which exhibits its highest electron transport capability.

The open circuit voltage (V_oc_) values of the **A1**–**A5** blends with **D1** have been calculated by using HOMO_D1_—LUMO_A1–A5_—0.3 equation. V_oc_ values are ranging from 1.04 to 1.50 eV. The Distribution of HOMO of donor and LUMO of acceptor has been studied for **D1**–**A4** blend which has provided information that the HOMO are resided on the donor polymer (**D1**) and LUMO are scattered on acceptor **A4**. Thus keeping in view the optoelectronic, molecular orbital distribution and reorganization energies **A3** and **A4** molecules were found a very good acceptor which can be synthesized as non-fullerene acceptor for future organic solar cell. These acceptors are expected to provide improved photovoltaic properties to OSCs.
